# Fatal Statin-Induced Rhabdomyolysis by Possible Interaction with Palbociclib

**DOI:** 10.3389/fonc.2017.00150

**Published:** 2017-07-17

**Authors:** Kellie Lynn Nelson, David Stenehjem, Meghan Driscoll, Glynn Weldon Gilcrease

**Affiliations:** ^1^Department of Internal Medicine and Neurology, University of Utah Medical Center, Salt Lake City, UT, United States; ^2^Department of Pharmacy Practice and Pharmaceutical Sciences, College of Pharmacy, University Minnesota Duluth, Duluth, MN, United States; ^3^Huntsman Cancer Institute, University of Utah Medical Center, Salt Lake City, UT, United States; ^4^Department of Pathology, University of Utah Medical Center, Salt Lake City, UT, United States; ^5^Department of Internal Medicine, Oncology Division, University of Utah Medical Center, Salt Lake City, UT, United States

**Keywords:** palbociclib, necrotizing rhabdomyolysis, progressive muscle weakness, CYP3A4, OATP1B1, atorvastatin, statins, statin-induced rhabdomyolysis

## Abstract

A 60- to 65-year-old female on prior statin therapy was initiated on palbociclib and fulvestrant for the treatment of metastatic, hormone-receptor positive breast cancer. She subsequently developed sudden progressive muscle weakness that progressed to death within weeks. The patient noticed progressive proximal muscle weakness after two cycles of palbociclib, with no other medication changes in the interim. This rapidly progressed and resulted in death within 7 days of presentation to hospital. There has been one previous report of rhabdmyolysis with palbociclib, occurring in a patient on concomitant statin. In this report, we discuss the possible aetiologies of this progressive rhabdomyolysis including time-dependent inhibition of CYP3A4 or inhibition of hepatic uptake transporters, e.g., OATP1B1.

## Introduction

Palbociclib is a highly selective oral inhibitor of cyclin-dependent kinases 4/6 (1, 2). Palbociclib combined with letrozole or fulvestrant is associated with significant improvements in progression-free survival in postmenopausal females with advanced hormone receptor positive, human epidermal growth factor receptor 2 (HER2) negative breast cancer. Since the publication of the PALOMA-2 and PALOMA-3 trials and subsequent FDA approvals, palbociclib use has grown considerably (3). The safety profile on any new drug may change in post-marketing experience due to new safety signals observed upon use in a larger patient population (4).

A commonly prescribed class of drugs in the general population for dyslipidemia is statins. There is conflicting evidence on the anticancer activity of statins, potentially prompting use. While retrospective case-control studies show a possible protective tendency with statin use in breast cancer, there are conflicting cohort studies refuting the protective association between cancer-specific mortality and statin use after cancer diagnosis (5, 6). Feared complications of statins are necrotizing myopathy and rhabdomyolysis; however, no fatal cases of myopathy were reported in PALOMA-2 and PALOMA-3 with pablociclib.

We describe a case of a 60- to 65-year-old woman on prior statin therapy that was initiated on palbociclib and fulvestrant and subsequently developed sudden, progressive muscle weakness, which progressed to death within weeks. Written and informed consent for this publication was obtained from the patient’s next of kin before publication of this case report.

## Case Report

A 60- to 65-year-old patient presented with a 7-day history of progressive generalized weakness. The patient was on atorvastatin 40 mg daily and had been on the drug for years. In early 2010s, the patient was diagnosed with estrogen receptor positive (ER+) and HER-2 negative, stage 1 (T1c N0 M0) breast cancer. The patient underwent mastectomy and 5 months of adjuvant cyclophosphamide, methotrexate, and 5-fluorouracil followed by anastrozole.

The patient represented with metastatic disease 1 year later with numerous liver, spleen, and bone metastases. The patient was treated with paclitaxel and gemcitabine. While the patient had initial response to combination chemotherapy, the patient showed progression of disease 1 year later with increased liver and bone metastases. Treatment with palbociclib and fulvestrant was started. The patient received their first dose of palbociclib 125 mg orally daily, 1 month after starting fulvestrant while continuing atorvastatin.

The patient received two cycles of palbociclib before presenting with 7 days of progressive proximal muscle weakness. The patient first noticed decreased stamina and pain in her proximal bilateral lower extremities when walking distances she previously could walk easily. This progressed to the bilateral proximal upper extremities. The proximal muscles weakness peaked 2 days before the patient’s hospitalization. The patient was unable to brush her hair or rise out of bed.

Exam showed diminished bilateral elbow extension and flexion, upper extremity abduction, adduction, flexion, and extension. The patient could move against gravity but was unable to move against resistance, and unable to flex their bilateral lower extremities at their hip bilaterally against gravity. The patient could extend and flex at the knee against gravity and had full range of motion against resistance of bilateral plantar and dorsiflexion. The patient’s reflexes were symmetric and intact throughout. Atorvastatin, palbociclib, and fulvestrant were discontinued at the time of the patient’s admission.

The patient had no evidence of systemic infection. There was no clear evidence of progressive disease in the liver by ultrasound or bone by MRI. MRI of the spine showed no evidence of cord compression. The patient’s creatinine kinase (CK) level on presentation was 14,572. Aldolase (59.1 U/L) and LDH (1,752 U/L) were additionally elevated. An antibody panel for myositis and myopathy was entirely negative (see Tables 1 and 2).

**Table 1:** Patient’s admission and last lab draw results.

	Admission	Day 6 of hospitalization
BUN	26 mg/dL	46 mg/dL
Creatinine	1.18 mg/dL	1.37 mg/dL
Protein, total	6.0 g/dL	4.9 g/dL
Albumin	2.6 g/dL	2.0 g/dL
Bilirubin, total	4.7 mg/dL	6.2 mg/dL
Alkaline phosphatase	897 U/L	1,224 U/L
AST/ALT	1,276/691 U/L	1,131/795 U/L
Creatinine kinase	14,572 U/L	10,686 U/L

**Table 2:** Patient’s myositis and myopathy laboratory results.

Test name	Results
Washington University’s neuromuscular antibody report (Serum)	GM1IgM 0, NP-9 IgM 9 (<3,000), NS6S IgM <15,000, GalNAc-GD1a IgM 0, asialo-GM1 IgM <3,000, GD1b IgM 0, GM1 IgG 0, GQ1b IgG 0, GD1b IgG 0, GalNac-GD1a IgG 0, Sulfatide IgG 0, Sulfatide IgM 0, MAG IgM 0, MAG Western blot negative, TS-HDS IgM < 10,000, FGFR3 IgG <3,000, Histone H3 IgM 0, GD1a IgM 0, Hu IgG (Western blot and IHC) negative, Yo IgG (Western blot and IHC) negative, CRMP-5 IgG negative, HMGCR IgG <2,500, Decorin IgM <2,500, Ho-1 IgG <3,000, NT5C1A IgG (Western Blot) negative, SRP IgG and IgM <3,000, MDA5 IgG negative, Heparan Sulfate IgM 0, Tubulin IgM 0, Tublin Western Blot negative, Neurofascin IgG <2,000, Contactin-1 IgG <2,000
HMGCR antibody, IgG	3 Units (negative)
ANA by IFA, IgG	<1:40
University of Utah Myositis Antibody Comprehensive Panel	SSA 52 IgG antibody negative, SSA 60 antibody IgG negative, ribonucleic protein IgG antibody negative, Mi-2 antibody negative, PL-7 (threonyl-tRNA synthetase) antibody negative, PL-12 (alanyl t-RNA synthetase) antibody negative, P155/140 (TIF1-gamma) antibody negative, Ku antibody negative, small nuclear RNP antibody negative, glycyl-tRNA synthetase antibody negative, signal recognition particle antibody negative, isoleucyl-tRNA synthetase antibody negative, PM/Scl complex antibodies

The patient continued to deteriorate clinically with increased weakness and pain in both the distal and proximal muscles. The patient was aggressively hydrated due to their increasing CK and kidney injury. A biopsy of the right vastus lateralis muscle was taken, and the patient was initiated on methylprednisolone. After one dose, the decision was made for comfort measures only. The patient died on their 8th day of hospitalization.

**Figure 1:**
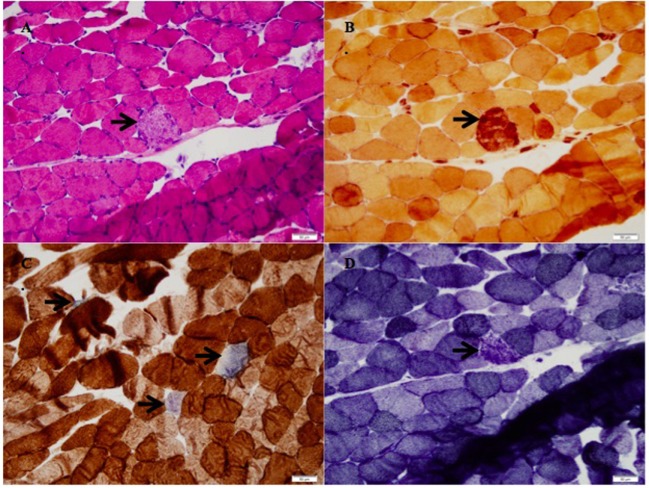
**(A)** H&E stain with necrotic fibers (arrow, 200× magnification), **(B)** stain for non-specific esterase with macrophages engulfing the necrotic myofiber (arrow, 200× magnification), **(C)** succinate dehydrogenase/cytochrome-C-oxidase (SDH/COX) combination stain with increased blue fibers, which lack cytochrome-C-oxidase function (arrows, 200× magnification), **(D)** DPNH stain with a nectoric fiber, but intact myotubular structures in the other myofibers (200× magnification).

The patient’s right vastus lateralis muscle biopsy showed the presence of scattered necrotic fibers in an otherwise quiescent background favoring a toxic or metabolic etiology. The necrotic fibers accounted for approximately 1% of the fibers in the biopsy without a lymphocytic or plasma cell inflammatory infiltrate in the muscle (Figure 1A). Macrophages within the myofibers were englufing the necrotic fibers (Figure 1B). This was the only inflammatory response identified and is the expected systemic response to myonecrosis. There was a mild increase in the number of myofibers whose mitochondrial respiratory functions were impaired or lost (Figure 1C). The DPNH stain shows a lack of damage to non-necrotic myofibers, which support the myonecrosis being an acute event. In chronic myopathic processes, the finding of moth-eaten fibers can be identified (Figure 1D).

## Discussion

This case of necrotizing rhabdomyolysis is notable for three factors. First, the timing of the patient’s progressive weakness coincided with starting palbociclib and fulvestrant. Second, the onset of symptoms and progression was rapid over the course of 1 week prior to hospitalization and the progressive decline did not improve with stopping the statin and palbociclib. Rather, the patient progressed quickly to death despite no clear evidence of progression of her breast cancer. Third, an extensive antibody workup was entirely negative despite the muscle biopsy showing necrotizing myopathy. While there has been one other reported case of palbociclib use with reported elevated LFTs and rhabdomylosis (this patient was also on a concomitant statin) in a Phase I trial in non-small cell lung cancer, this patient survived and the symptoms improved quickly after stopping palbociclib (7).

We hypothesize that there was additive or symbiotic toxicity of palbociclib and atorvastatin, or the interaction of palbociclib and atorvastatin precipitated statin-induced rhabdomyolysis. One prominent histologic features in statin-induced rhabdomyolysis muscle-biopsy specimens is myofiber necrosis (8, 9). Her HMG-CoA reductase IgG was negative, which may be due to the rapidity of the patient’s disease. It is also possible that a non-immune-mediated process occurred in the setting of altered metabolism such as direct toxicity to the myofibers by the statin and/or palbociclib.

The patient’s decline coincided with the initiation of palbociclib and fulvestrant. The patient had elevated AST > ALT after starting palbociclib, which may have been from muscle injury. The patient had been maintained on atorvastatin 40 mg daily for years prior to beginning the palbociclib. The patient was not on any strong inhibitors or inducers of CYP3A4, and no other new medications were started prior to their progressive myopathy. Fulvestrant is a CYP3A4 substrate, however, CYP3A4 inhibitors or inducers do not alter pharmacokinetics.

Approximately 60% of cases of statin-induced rhabdomyolysis are due to concomitant administration of a CYP3A4 inhibitor (8–10). Additionally, a dose–response relationship exists with higher statin doses resulting in increased frequency of rhabdomyolysis (11). Atorvastatin undergoes extensive pre-systemic clearance and first-pass metabolism in the liver by CYP3A4, with a subsequent 12% oral bioavailability (12). Drugs with a high pre-systemic elimination and low bioavailability may lead to supratherapeutic concentrations if metabolism is inhibited (12). Atorvastatin is extensively metabolized by CYP3A4 (8).

Palbociclib is a time-dependent inhibitor of CYP3A4 and is classified as a weak inhibitor. In the clinical drug–drug interaction studies, palbociclib at 125 mg daily increased midazolam (sensitive CYP3A4 substrate) mean C_max_ by 37% and AUC values by 61% compared to midazolam alone (13). Therefore, in this case, the addition of palbociclib to atorvastatin may have resulted in a clinically significant increase in atorvastatin concentration *via* palbociclib CYP3A4-mediated inhibition resulting in statin-induced rhabdomyolysis and eventually death.

Another potential mechanism of statin-associated myopathy is through inhibition of hepatic uptake transporters (i.e., OATP1B1) or genetic variation in *SLCO1B1* (8, 14). Hepatic uptake transporter inhibition may result in increased statin concentrations and may precipitate myopathy and rhabdomyolysis. However, both palbociclib and fulvestrant are not reported to inhibit these influx transport mechanisms. It is unknown if the patient had a genetic variant in *SLCO1B1* that would have affected atorvastatin serum concentrations.

## Conclusion

While our patient is the second case report published about the possibility of a drug interaction between a statin and palbociclib causing necrotizing rhabdomyolysis, our case was notable for its quick onset and progression to death despite stopping atorvastatin and palbociclib. The benefit of continuing a statin should be weighed against the risk of the patient having a cardiovascular event within this short-term period. If statins are prescribed concomitantly with palbociclib, careful monitoring of AST/ALT and CK is warranted.

## Author Contributions

KN was the primary author who authored all drafts of the paper and gathered the clinical information regarding the patient. DS was an editing author and provided the critical pharmacologic knowledge to base our hypothesis regarding the etiology of the patient’s condition. MD provided several edits to the manuscript and also provided the pathology input and pathology slides of the muscle biopsy. GG was the supervising attending physician who oversaw the patient’s care and additionally organized the authors so that the paper could come together and have the correct pathology descriptions and pharmacologic basis. All authors have read and approve the final version of the manuscript.

## Conflict of Interest Statement

The authors declare that the research was conducted in the absence of any commercial or financial relationships that could be construed as a potential conflict of interest.
